# Description of the molecular and phenotypic spectrum of Wiedemann-Steiner syndrome in Chinese patients

**DOI:** 10.1186/s13023-018-0909-0

**Published:** 2018-10-11

**Authors:** Niu Li, Yirou Wang, Yu Yang, Pengpeng Wang, Hui Huang, Shiyi Xiong, Luming Sun, Min Cheng, Cui Song, Xinran Cheng, Yu Ding, Guoying Chang, Yao Chen, Yufei Xu, Tingting Yu, Ru-en Yao, Yiping Shen, Xiumin Wang, Jian Wang

**Affiliations:** 1 Department of Medical Genetics and Molecular Diagnostic Laboratory, Shanghai Children’s Medical Center, Shanghai Jiaotong University School of Medicine, Shanghai, 200127 China; 2 Institute of Pediatric Translational Medicine, Shanghai Children’s Medical Center, Shanghai Jiao Tong University School of Medicine, Shanghai, 200127 China; 3 Department of Endocrinology and Metabolism, Shanghai Children’s Medical Center, Shanghai Jiaotong University School of Medicine, Shanghai, 200127 China; 4 Department of Endocrinology, Metabolism, and Genetics, Jiangxi Provincial Children’s Hospital, Nanchang, 330029 Jiangxi China; 5 MyGenostics Inc., Beijing, 101318 China; 6 Central laboratory, Jiangxi Provincial Children’s Hospital, Nanchang, 330029 Jiangxi China; 7 Fetal Medicine Unit & Prenatal diagnosis center, Shanghai First Maternity and Infant hospital, Tongji University School of Medicine, Shanghai, People’s Republic of China; 8 Department of Neurology, Children’s Hospital of Chongqing Medical University, Chongqing, 400014 China; 9 Department of Endocrinology and Genetic Metabolic Diseases, Ministry of Education Key Laboratory of Child Development and Disorders, China International Science and Technology Cooperation Base of Child Development and Critical Disorders. Chongqing Key Laboratory of Pediatrics, Children’s Hospital of Chongqing Medical University, Chongqing, 400014 China; 10 Department of Endocrinology and Metabolism, Chengdu Women’s and Children’s Central Hospital, Sichuan Province, Chengdu, 610091 China; 11 Division of Genetics and Genomics, Boston Children’s Hospital, Harvard Medical School, Boston, MA 02115 USA; 12 Shanghai Children’s Medical Center, Shanghai Jiaotong University School of Medicine, 1678 Dongfang Road, Shanghai, 200127 People’s Republic of China

**Keywords:** Wiedemann–Steiner syndrome, KMT2A variation, Chinese patients, Phenotypic differences

## Abstract

**Background:**

Wiedemann–Steiner syndrome (WDSTS) is a rare genetic disorder characterized by facial gestalt, neurodevelopmental delay, skeletal anomalies and growth retardation, which is caused by variation of *KMT2A* gene. To date, only 2 Chinese WDSTS patients have been reported. Here, we report the phenotypes and *KMT2A* gene variations in 14 unrelated Chinese WDSTS patients and investigate the phenotypic differences between the Chinese and French cohorts.

**Methods:**

Next generation sequencing was performed for each patient, and the variants in the *KMT2A* gene were validated by Sanger sequencing. The phenotypes of 16 Chinese WDSTS patients were summarized and compared to 33 French patients.

**Results:**

Genetic sequencing identified 13 deleterious de novo *KMT2A* variants in 14 patients, including 10 truncating, 2 missenses and 1 splicing variants. Of the 13 variants, 11 are novel and two have been reported previously. One of the patients is mosaic in the *KMT2A* gene. The variation spectra and phenotypic profiles of the Chinese WDSTS patients showed no difference with patients of other ethnicities; however, differ in the frequencies of several clinical features. We demonstrated that variations in the *KMT2A* gene can lead to both advanced and delayed bone age. We identified 6 novel phenotypes, which include microcephaly, deep palmar crease, external ear deformity, carpal epiphyseal growth retardation, dyslipidemia, and glossoptosis. In addition, patients harbored missense variants in the CXXC zinc finger domain of *KMT2A* showed more severe neurophenotypes.

**Conclusion:**

Our study consists of the largest cohort of Chinese WDSTS patients that continues to expand the WDSTS phenotypic and variation spectrum. Our results support the notion that the CXXC zinc finger domain of *KMT2A* gene is a hotspot for missense variants associated with more severe neurophenotypes.

**Electronic supplementary material:**

The online version of this article (10.1186/s13023-018-0909-0) contains supplementary material, which is available to authorized users.

## Background

Wiedemann–Steiner syndrome (WDSTS, OMIM #605130) is a pleiotropic and extremely rare autosomal dominant disorder, which was first reported by Wiedemann et al. in 1989 and later described by Steiner et al. in 2000 [1]. Using whole-exome sequencing (WES), Jones et al. revealed that germline heterozygous variations of the *KMT2A* gene (OMIM #159555) was responsible for WDSTS in five patients in 2012 [2]. The *KMT2A* gene, also known as *MLL*, encodes a histone lysine methyltransferase that plays a critical role in regulating gene expression during early development and hematopoiesis [3]. Because KMT2A regulates multiple *Hox* and *Wnt* genes through histone H3 lysine 4 (H3K4) methylation [4], phenotypes of the WDSTS patients are complex and involve multiple systems, including facial features, skeletal development, and neuro development. A recent summary study pointed out that the clinical features of developmental delay (DD), intellectual disability (ID), postnatal growth retardation, palpebral fissures, down-slanted, wide nasal bridge, broad nasal tip, long eyelashes, and thick eyebrows are more common in WDSTS patients [5]. Increasing number of WDSTS patients of different ethnicities are being identified due to the rapid advance of DNA sequencing technologies, which continues to expand the phenotype spectra with novel features [5]. To date, at least 69 WDSTS patients with variations in *KMT2A* gene were confirmed, of whom only two are Chinese [5–11]. Though most of the features in the two Chinese patients are similar to patients of other ethnicities, whether the absent palmar proximal transverse creases is a unique feature of Chinese patients and whether there are novel phenotypes in Chinese patients remain unclear. Meanwhile, our knowledge on the phenotype spectrum and variation spectrum of Chinese patients are still very limited.

Here, we studied the phenotypes and *KMT2A* variations of 14 unrelated Chinese WDSTS patients. We identified 13 de novo heterozygous variants in the *KMT2A* gene, including 10 truncating, 2 missense, and 1 splicing variants. Of them, 11 are novel. The phenotypes of Chinese WDSTS patients were compared with a French cohort. In addition, we hereby report several novel clinical features of WDSTS, including macrocephaly, deep palmar crease, external ear deformity, carpal epiphyseal growth retardation, dyslipidemia, and glossoptosis.

## Methods

### Patients

A total of 14 Chinese patients (8 females and 6 males), aged from 1.5 years to 25 years old, were enrolled from Shanghai Children’s Medical Center, Jiangxi Provincial Children’s Hospital, Shanghai First Maternity and Infant Hospital, Chongqing Children’s Hospital, and Chengdu Women’s and Children’s Central Hospital in China. All patients’ parents are unaffected and non-consanguineous in our study.

### Next generation sequencing (NGS)

For Patients 1–7, 10 and 12, proband-only targeted-NGS using inherited disease panel (including 2742 disease-causing genes, cat No.5190–7519, Agilent technologies Inc., Santa Clara, CA, USA) was performed in Shanghai Children’s Medical Center as described previously [12]. For Patients 8, 9, 11 and 14, proband-only targeted NGS was performed by a commercial company (MyGenostics, Inc., Beijing, China), using a clinical exome capture panel containing 4231 disease-causing genes. For patient 13, trio-based WES was performed by the short stature sequencing program [13]. Variants detected by NGS were confirmed by Sanger sequencing in each patient and their parents, when the samples were available.

### Statistics analysis

The statistical analysis between two cohorts was performed by Chi-square (χ2) test using the SPSS 17.0 (Statistical Package for the Social Sciences Inc., Chicago, IL, USA). In data statistics, *p* < 0.05 is considered of suggestive significance.

## Results

### *KMT2A* variants

We identified 13 different *KMT2A* variants (10 truncating, 2 missense, 1 splicing) (Table 1 and Additional file 1: Table S1), with Patient 3 and Patient 7 harboring the same truncating variant (p.Ser774Valfs*12). The two missense variants occurred in the CXXC zinc finger domain (p.Gly1168Asp) and the SET domain (p.Arg3906Cys), respectively (Additional file 2: Table S2). Multiple in silico tools predict deleterious outcomes of these two missense variants (p.Gly1168Asp: scored 0.000 in SIFT, 1.0 in Polyphen-2, 1.0 in MutationTaster, and 35.0 in CADD; p.Arg3906Cys: scored 0.002 in SIFT, 1.0 in Polyphen-2, 1.0 in MutationTaster, and 29.3 in CADD). Of the 13 identified variants, 11 are novel, and the p.Gly1168Asp and p.Ser774Valfs*12 variants have been reported previously [11, 14]. Except the variants identified in patient 9, whose mothers’ sample was not available, the other 12 variants were confirmed to be de novo. According to the variant-interpretation guidelines from the American College of Medical Genetics and Genomics and the Association for Molecular Pathology [15], the 2 missense variants were classified as likely pathogenic, and the others are pathogenic. In additional, a total of 106 X of the base at c.5871 in Patient 10 was sequenced. Interestingly, the variant allele (A) has much lower proportion (24 X, 22.6%) when compared to the wild-type allele (T) (82 X, 77.4%), which is confirmed by Sanger sequencing (Additional file 3: Figure S1), indicating mosaicism in the patient.

**Table 1:** Clinical Summaries of the Chinese Wiedemann–Steiner Syndrome (WDSTS) Patients

Patient ID	1	2	3	4	5	6	7		8
Gender	F	M	M	F	M	F	F		F
Gestation (weeks)	35	Full term	Full term	35	Full term	Full term	Full term		Full term
Birth height (cm)/weight (kg)	49/2.9	Unknown/3.1	50/3.2	43/1.9	50/3.2	Unknown/3.4	Unknown/3.3		Unknown/3.8
Age at last examination	3-year-3-month	3-year	6-year-7-month	5-year	5-year-7-month	8-year-10-month	25-year		9-year-10-month
Current height (cm)/weight (kg)	92 (−1.75 SD)/ 11.8 (−2.30 SD)	85(−3.16 SD)/ 11.0(− 2.67 SD)	106 (− 3.18 SD)/ 16.2 (− 2.51SD)	96.5 (−5.32 SD)/ 13.5 (− 4.12 SD)	105(−2.26 SD)/ 18.0 (− 1.09 SD)	131 (−0.34 SD)/ 32.5 (+ 1.01 SD)	158 (− 0.57 SD)/ 65 (+ 1.68 SD)		107 (− 4.72 SD)/ 17.0(− 1.90 SD)
Craniofacial features									
Microcephaly (HPO:0000252)	+	+	+	–	–	–	–		–
Macrocephaly (HPO:0000256)	–	–	–	–	–	–	+		–
Prominent forehead (HPO:0011220)	+	+	–	–	+	+	–		–
Hypertelorism (HPO:0000316)	+	+	+	+	+	+	–		+
Ptosis (HPO:0000508)	–	+	+	–	–	–	+		+
Epicanthus (HPO:0000286)	–	–	–	–	–	–	–		–
Down-turned palpebral fissures (HPO:0000494)	+	+	+	+	+	–	–		+
Wide nasal bridge (HPO:0000431)	+	+	–	+	+	+	–		+
Depressed nasal bridge (HPO:0005280)	+	+	–	+	–	+	–		+
Long philtrum (HPO:0000343)	–	+	+	–	+	+	–		–
Low set ears (HPO:0000368)	–	+	+	+	–	–	–		–
External ear deformity(HPO:0040111)	–	–	–	–	–	–	–		–
Thin upper lip (HPO:0000219)	+	+	+	–	+	–	–		–
Down-turned corners of the mouth (HPO:0000153)	+	+	+	+	+	–	–		+
Micrognathia (HPO:0000347)	–	–	+	+	+	+	–		–
Skeletal anomalies									
Advanced bone age (HPO:0200001)	nd	nd	nd	–	+	+	nd		–
Delayed bone age (HPO:0003799)	nd	nd	nd	+	–	–	nd		+
Brachydactyly (HPO:0001156)	–	–	–	+	+	+	+		–
Syndactyly (HPO:0001159)	–	–	–	–	–	–	–		–
Clinodactyly (HPO:0030084)	–	–	–	–	+	+	+		–
Puffy hands and feet	–	–	–	+	–	–	+		+
Small hands and feet	–	–	–	+	–	–	+		+
Carpal epiphyseal growth retardation	nd	nd	nd	–	–	–	–		–
Scoliosis (HPO:0002650)	–	–	–	–	–	–	–		–
Sacral dimple (HPO:0000960)	–	–	–	–	–	–	–		+
Absent palmar proximal transverse creases (HPO:0010489)	–	–	–	–	–	–	–		–
Deep palmar crease (HPO:0006191)	–	–	–	–	–	–	–		–
Hairiness									
Thick hair (HPO:0100874)	+	+	+	–	–	+	+		+
Thick eyebrows (HPO:0000574)	–	–	–	–	–	+	+		+
Synophrys (HPO:0000664)	–	–	–	–	–	–	+		–
Arched eyebrows (HPO:0002553)	–	+	–	–	+	–	+		–
Long eyelashes (HPO:0000527)	+	+	+	+	+	+	–		+
Low hair line (HPO:0000294)	+	+	+	+	+	–	+		+
Hypertrichosis, cubiti (HPO:0000998)	–	–	–	–	–	+	+		+
Hypertrichosis, back (HPO:0000998)	mild	mild	–	+	mild	mild	+		mild
Hypertrichosis, lower limbs (HPO:0000998)	–	–	–	–	–	–	–		+
Developmental and neurology									
Walking delay (HPO:0031936)	–	+	–	–	+	–	–		–
Language delay (HPO:0000750)	+	+	+	–	+	–	+		+
Intellectual disability (HPO:0001249)	mild	+	+	nd	+	–	+		+
Aggressive behavior (HPO:0000718)	–	+	+	–	+	–	–		–
Hyperactivity (HPO:0007018)	–	–	–	–	+	–	–		+
Autism (HPO:0000717)	–	–	–	–	+	–	–		–
Organic problems									
Strabismus (HPO:0000486)	–	–	–	–	–	–	–		–
Hyperopia (HPO:0008499)	–	–	+	–	–	–	–		–
High palate (HPO:0000218)	–	–	+	+	+	+	+		+
Cleft palate (HPO:0000175)	+	–	–	–	–	–			–
Glossoptosis (HPO:0000162)	–	–	–	–	–	–	–		–
Feeding difficulties (HPO:0011968)	+	–	–	–	+	–	–		–
Cardiac anomaly	–	–	–	–	PDA	–	–		–
Dyslipidemia	–	–	–	–	–	–	–		–
Abnormality of the teeth (HPO:0000164)	–	–	+	–	+	–	–		+
GH deficiency (HPO:0000824)	ne	ne	+	+	ne	ne	ne		ne
*KMT2A* variant	p.Pro1281Leufs*75	p.Gly3585Argfs*8	p.Ser774Valfs*12	p.Arg3906Cys	p.Gly1168Asp	p.Arg1081*	p.Ser774Valfs*12		c.10900 + 2 T > C
	This study						Sun et al.2017 (ref 6)		Chinese patients (*N* = 16; %)
Patient ID	9	10	11	12	13	14	A.II-5	B.II-1	
Gender	M	M	F	M	F	F	M	M	8 M/8F
Gestation (weeks)	Full term	Full term	38^+ 5^	Full term	35^+ 5^	38^+ 5^	nd	nd	
Birth height (cm)/weight (kg)	Unknown	Unknown/2.6	51/2.9	50/2.9	46/2.5	Unknown /2.3	nd	nd	
Age at last examination	12-year	6-year-9-month	18-month	4-year	17-month	20-month	3-year	6-year	
Current height (cm)/weight (kg)	118 (−3.16 SD)/ 19.5 (−3.76 SD)	102.8 (−4.18 SD) /15.7 (− 3.06 SD)	72 (− 3.28 SD) / 8.0 (−2.75 SD)	100.5 (−0.89 SD)/ 15.4 (0.47 SD)	66.0 (−6.25 SD)/ 6.7 (− 4.13 SD)	66.0 (−5.90 SD) / 7.1 (− 4.16 SD)	<-2SD/nd	<-2SD/nd	
Craniofacial features									
Microcephaly (HPO:0000252)	+	+	–	–	–	+	+	+	8/16; 50%
Macrocephaly (HPO:0000256)	–	–	–	–	–	–	–	–	1/16; 6%
Prominent forehead (HPO:0011220)	+	–	+	–	+	–	–	–	7/16; 44%
Hypertelorism (HPO:0000316)	–	–	+	+	+	+	+	+	13/16; 81%
Ptosis (HPO:0000508)	+	–	+	+	–	+	+	+	10/16; 63%
Epicanthus (HPO:0000286)	–	+	–	–	–	–	–	+	2/16; 13%
Down-turned palpebral fissures (HPO:0000494)	+	–	+	+	+	–	+	+	12/16; 75%
Wide nasal bridge (HPO:0000431)	–	–	+	+	–	+	+	–	10/16; 63%
Depressed nasal bridge (HPO:0005280)	–	–	+	+	–	–	+	+	9/16; 56%
Long philtrum (HPO:0000343)	–	–	+	+	+	–	+	+	9/16; 56%
Low set ears (HPO:0000368)	–	+	–	+	–	–	+	–	6/16; 38%
External ear deformity (HPO:0040111)	–	+	–	+	–	–			2/16; 13%
Thin upper lip (HPO:0000219)	+	+	+	+	–	–	+	+	8/16; 50%
Down-turned corners of the mouth (HPO:0000153)	–	–	+	+	+	–	+	+	13/16; 81%
Micrognathia (HPO:0000347)	–	–	–	–	+	–			7/16; 44%
Skeletal anomalies									
Advanced bone age (HPO:0200001)	–	–	–	–	nd	nd	–	–	2/10; 20%
Delayed bone age (HPO:0003799)	+	+	+	–	nd	nd	+	+	7/10; 70%
Brachydactyly (HPO:0001156)	–	+	–	+	–	+	–	+	8/16; 50%
Syndactyly (HPO:0001159)	2–3 toe	2–3 toe	–	–	–	2–3 toe	–	–	3/16; 19%
Clinodactyly (HPO:0030084)	–		–	+	–	–	–	–	4/16; 25%
Puffy hands and feet	–	+	–	+	+	–	+	+	8/16; 50%
Small hands and feet	–	+	+	+	–	–	+	+	8/16; 50%
Carpal epiphyseal growth retardation	–	+	–	–	–	–			1/13; 8%
Scoliosis (HPO:0002650)	–	+	–	–	–	–	–	–	1/16; 6%
Sacral dimple (HPO:0000960)	+	+	–	–	–	–	+	–	4/16; 25%
Absent palmar proximal transverse creases (HPO:0010489)	–	–	–	–	–	–	+	+	2/16; 13%
Deep palmar crease (HPO:0006191)	+	–	–	–	–	–	–	–	1/16; 6%
Hairiness									
Thick hair (HPO:0100874)	+	+	+	+	+	+	+	+	14/16; 88%
Thick eyebrows (HPO:0000574)	–	+	+	–	–	+	–	–	6/16; 38%
Synophrys (HPO:0000664)	–	–	–	–	–	–	–	–	1/16; 6%
Arched eyebrows (HPO:0002553)	–	+	+	–	–	+	–	–	6/16; 44%
Long eyelashes (HPO:0000527)	+	+	+	+	–	+	+	+	15/16; 94%
Low hair line (HPO:0000294)	+	+	+	–	–	+	+	+	13/16; 81%
Hypertrichosis, cubiti (HPO:0000998)	+	+	mild	mild	–	–	–	–	7/16; 44%
Hypertrichosis, back (HPO:0000998)	+	+	–	mild	mild	–	+	–	12/16; 75%
Hypertrichosis, lower limbs (HPO:0000998)	+	+	–	+	–	+	+	+	8/16; 50%
Developmental and neurology									
Walking delay (HPO:0031936)	+	+	+	+	+	+	+	+	10/16; 63%
Language delay (HPO:0000750)	+	+	too young	+	too young	+	+	+	12/14; 86%
Intellectual disability (HPO:0001249)	+	+	+	+	+	+	+	+	14/15; 93%
Aggressive behavior (HPO:0000718)	–	–	–	+	–	–	–	–	4/16; 25%
Hyperactivity (HPO:0007018)	–	–	–	–	–	–	–	–	2/16; 13%
Autism (HPO:0000717)	–	–	–	–	–	–	–	–	1/16; 6%
Organic problems									
Strabismus (HPO:0000486)	+	+	–	–	too young	too young	–	+	3/14; 21%
Hyperopia (HPO:0008499)	–	–	–	–	too young	too young	–	–	1/14; 7%
High palate (HPO:0000218)	+	+	–	+	+	+	–	+	12/16; 75%
Cleft palate (HPO:0000175)	–	–	–	–	+	–	–	–	1/16; 6%
Glossoptosis (HPO:0000162)	–	–	–	–	+	–	–	–	1/15; 7%
Feeding difficulties (HPO:0011968)	–	–	–	–	+	–	+	+	5/16; 31%
Cardiac anomaly	–	BAV	–	–	–	–	PDA	–	3/16; 19%
Dyslipidemia	–	+	–	–	–	–	–	–	1/16; 6%
Abnormality of the teeth (HPO:0000164)	+	+	–	+	+	–	–	–	7/16; 44%
GH deficiency (HPO:0000824)	ne	+	ne	ne	ne	ne	nd	nd	3/3; 100%
*KMT2A* variant	p.Gln3613*	p.Tyr1957*	Pro1354Leufs*2	p.Glu2018fs*7	p.Arg301*	p.Trp838lfs*9	p.Gln2803*	p.Gln819*	

*F* female, *M* male, *SD* standard deviation, *HPO* Human phenotype ontology, *nd* no data, *ne* not evaluated, *PDA* patent ductus arteriosus, *BAV* bicuspid aortic valve, *GH* Growth hormone

### Clinical presentation

**Fig. 1:**
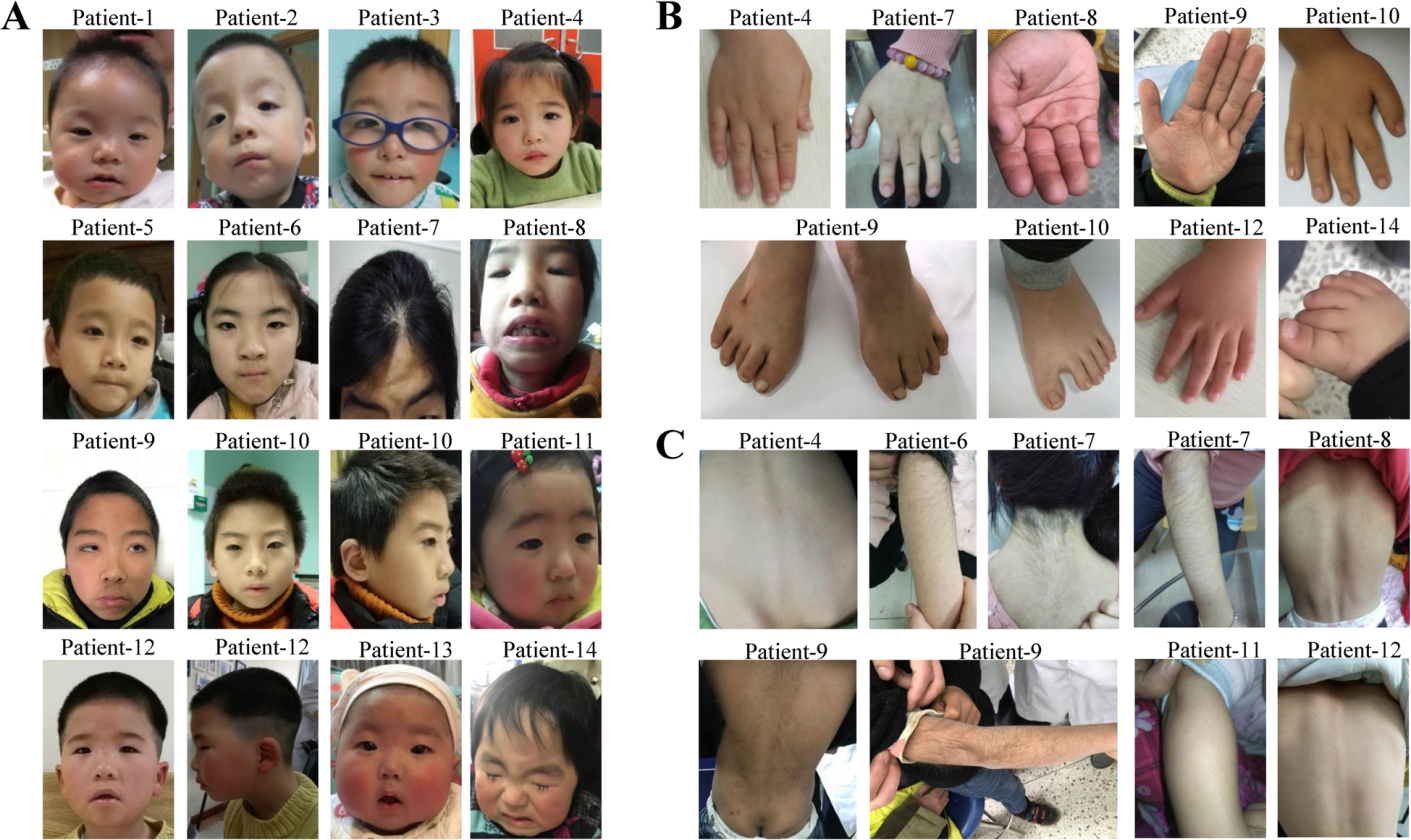
Phenotypes of the patients. **a** Facial appearances of patients 1–14. Patients 10 and 12 showed external ear deformity. **b** Malformations of hands and feet in seven patients. **c** Hypertrichosis of seven patients

**Fig. 2:**
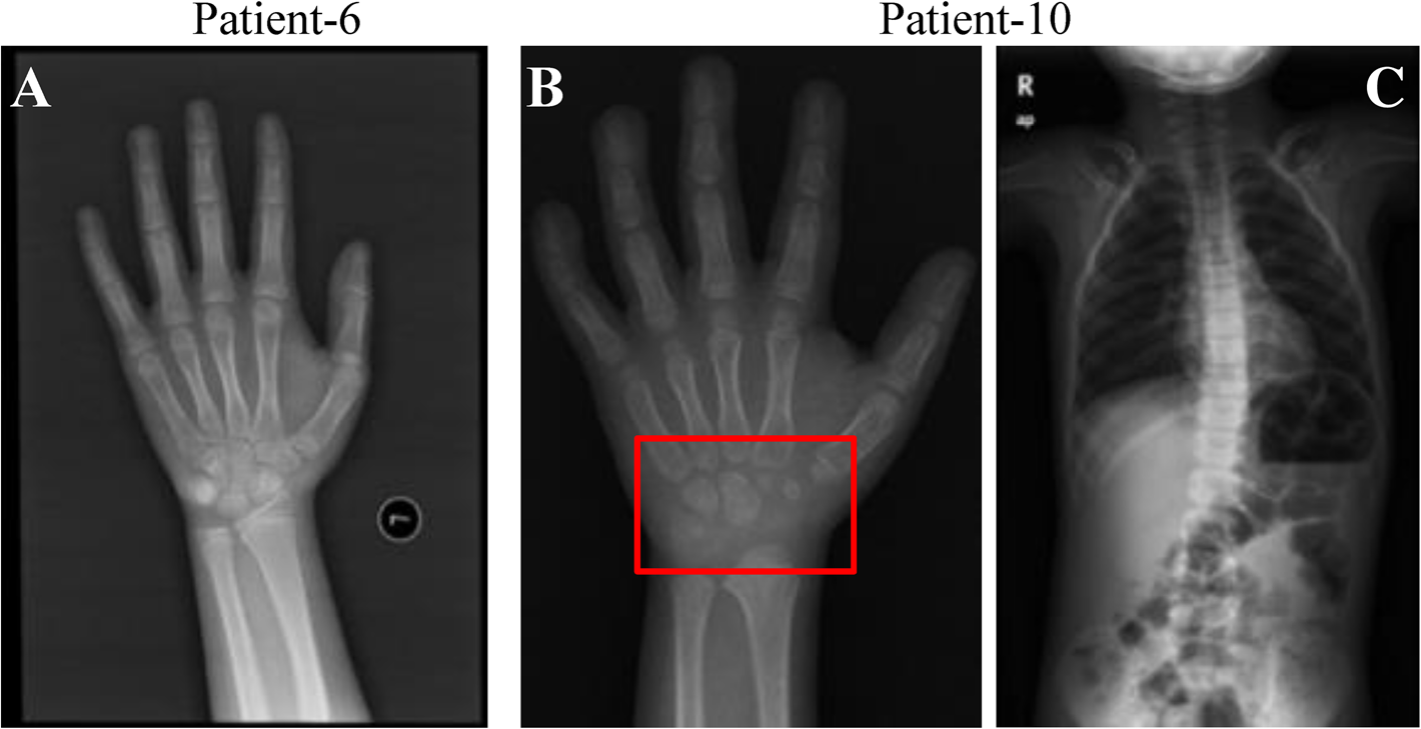
X-ray results of Patients 6 and 10. **a** The bone age of Patient 6 was advanced for 2.5 years. Patient 10 had a bone age of 4.5–5 years old and severe carpal epiphyseal growth retardation (**b**) and scoliosis (**c**)

To fully describe the clinical features of Chinese WDSTS patients, 2 previously reported patients [6] were included in our analysis, to make the sample size 16. A comprehensive list of clinical presentation is summarized in Table 1, and Figs. 1 and 2.

In summary, the most frequent (with frequency ≥ 70%) clinical features in Chinese WDSTS patients were as follows: long eyelashes, ID, thick hair, delay in language development, low hair line, hypertelorism, downturned corners of the mouth, down-turned palpebral fissures, vertically narrow palpebral fissures, high palate, hypertrichosis on back, postnatal growth retardation for height (12/16, 75%), and delayed bone age.

Additionally, six clinical features were reported in WDSTS patients for the first time, including macrocephaly in Patient 7, external ear deformity in Patients 10 and 12 (Fig. 1a), deep palmar crease in Patient 9 (Fig. 1b), carpal epiphyseal growth retardation in Patient 10 (Fig. 2b), dyslipidemia in Patient 10 with mild increased in cholesterol level (5.78 mmol/L, normal range: 2.50–5.20 mmol/L) and low-density in lipoprotein level (4.18 mmol/L, normal range: 0–3.36 mmol/L), and glossoptosis in Patient 13.

### Intervention

Patient 1 received rehabilitation training to improve the gross motor development before 2 years old. Patient 3 received growth hormone (GH) therapy (0.15 IU/kg/d = 0.05 mg/kg/d) for 14 months and had a 12 cm gain in height (0.85 cm/month).The GH treatment effect of Patient 3 is similar with a recently reported WDSTS patient, whose height velocity increased to 13.3 cm/year [16]. Patient 6 was initially diagnosed with puberty variation with bilateral breast development for the past 5 months at 8-year-10-minth old. She was therefore treated with gonadotrophin releasing hormone (GnRH) analogue to inhibit the rapid sexual development, hoping to improve her adult height. The latest evaluation of Patient 6 at 9-year-4-month old showed well-controlled of the breast development and the normal levels of sex hormones, 17-hydroxyprogesterone, adrenocorticotropic hormone, and dehydroepiandrosterone sulfate. Patient 11 is currently undergoing rehabilitation training to improve her gross motor development. Patient 7 was naturally pregnant with triplets and thus she was provided with genetic counseling. Further examination revealed that one of the three fetuses had scoliosis and one had pulmonary artery atresia. The family decided to take fetal reduction surgery to remove the fetus with scoliosis. A recent follow-up showed that the other two babies were born, and the normal baby was confirmed to carry wild-type *KMT2A* gene by genetic testing. Unfortunately, we were not able to get genetic information of the baby who had pulmonary artery atresia, since the parents refused to have genetic testing on the baby. Patient 13 had breathing difficulty after birth due to glossoptosis and micrognathia, and thus he underwent a mandibular traction surgery at 50 days of age.

### Phenotypic comparison of the Chinese and French cohorts

For further understand WDSTS, we did statistical analysis for the phenotypes between the 16 Chinese patients and a cohort of 33 French patients [11]. As shown in Table 2, no significant differences of age span, gender distribution, and the *KMT2A* gene variant spectrum in the two cohorts. The overall clinical feature profiles are similar in these two groups, however, several differences still can be observed. Three features of ptosis, thick eyebrows, and feeding difficulties showed significant statistical difference in the two cohorts. The Chinese cohort had higher frequency of ptosis but lower frequency of thick eyebrows and feeding difficulties. In addition, though no statistical difference was observed, postnatal growth retardation, microcephaly, down-turned palpebral fissures, long eyelashes, brachydactyly, and delayed bone age were more frequent in the Chinese cohort, while the frequency of thin upper lip, hyperopia, and congenital heart malformations were higher in the French cohort.

**Table 2:** Clinical features compared within Chinese and French WDSTS patients

	Chinese cohort (*N* = 16; %)	French cohort (*N* = 33; %) (Ref. 11)	*P* value (χ2 test)
General information			
Gender	F8 (50%)/M8 (50%)	F11 (33%)/M22 (67%)	0.261
Age at last examination (years)	1.5 to 25	3 to 36	
Postnatal growth retardation (H)	12/16 (75%)	15/32 (47%)	0.064
Postnatal growth retardation (W)	9/14 (64%)	11/30 (37%)	0..087
Postnatal growth retardation (H + W) (HPO:0008897)	8/14 (57%)	9/30 (30%)	0.085
*KMT2A* variants	15 different variants	29 different variants	0,639
Nonsense variants	6	8	
Frameshift variants	6	12	
Missense variants	2	8	
Splicing variants	1	1	
Craniofacial features			
Microcephaly (HPO:0000252)	8/16 (50%)	10/30 (33%)	0.270
Hypertelorism (HPO:0000316)	13/16 (81%)	21/32 (66%)	0.432
Ptosis (HPO:0000508)	10/16 (63%)	5/32 (16%)	0.001
Down-turned palpebral fissures (HPO:0000494)	12/16 (75%)	18/31 (58%)	0.252
Wide nasal bridge (HPO:0000431)	10/16 (63%)	22/31 (71%)	0.555
Long philtrum (HPO:0000343)	9/16 (56%)	20/32 (63%)	0.676
Low set ears (HPO:0000368)	6/16 (38%)	15/30 (50%)	0.418
Thin upper lip (HPO:0000219)	8/16 (50%)	24/32 (75%)	0.083
Skeletal anomalies			
Advanced bone age (HPO:0200001)	2/10 (20%)	7/15 (47%)	0.229
Delayed bone age (HPO:0003799)	7/10 (70%)	5/15 (33%)	0.111
Brachydactyly (HPO:0001156)	8/16 (50%)	9/29 (31%)	0.209
Clinodactyly (HPO:0030084)	4/16 (25%)	6/28 (21%)	1.000
Sacral dimple (HPO:0000960)	4/16 (25%)	8/25 (32%)	0.631
Hairiness			
Thick eyebrows (HPO:0000574)	6/16 (38%)	23/29 (79%)	0.005
Long eyelashes (HPO:0000527)	15/16 (94%)	24/32 (75%)	0.239
Hypertrichosis, cubiti (HPO:0000998)	7/16 (44%)	19/31 (61%)	0.252
Hypertrichosis, back (HPO:0000998)	12/16 (75%)	21/31 (68%)	0.858
Hypertrichosis, lower limbs (HPO:0000998)	8/16 (50%)	9/24 (38%)	0.433
Developmental and neurology			
Walking delay (HPO:0031936)	10/16 (63%)	19/31 (61%)	0.936
Language delay (HP:0000750)	12/14 (86%)	24/30 (80%)	0.970
Intellectual disability (HPO:0001249)	14/15 (93%)	33/33 (100%)	0.683
Aggressive behavior (HPO:0000718)	4/16 (25%)	4/31 (13%)	0.525
Organic problems			
Strabismus (HPO:0000486)	3/14 (21%)	7/32 (22%)	1.000
Hyperopia (HPO:0008499)	1/14 (7%)	9/32 (28%)	0.230
Cardiac anomaly	3/16 (19%)	8/22 (36%)	0.412
Feeding difficulties (HPO:0011968)	5/16 (31%)	20/31 (65%)	0.030

*F* female, *M* male, *H* height, *W* weight, *HPO* Human phenotype ontology

## Discussion

In this study, we recruited 14 unrelated Chinese WDSTS patients. To our knowledge, this is the largest cohort of Chinese WDSTS patients with the greatest age span (from pediatric to adult) that have been reported. Recently, Sun et al. reported two Chinese male patients who presented with absent palmar proximal transverse creases, which may be a unique characteristic of Chinese WDSTS patients [6]. We thus examined all the 14 patients and found that no patients had abnormal palmar crease except for Patient 9, who had deep palmar crease (Fig. 1c). We speculate that the lack of abnormal palmar crease in patients of other ethnicities may be a result of negligence during phenotype assessment. Additionally, we revealed several novel clinical features in WDSTS patients, including macrocephaly, deep palmar crease, external ear deformity, carpal epiphyseal growth retardation, dyslipidemia, and glossoptosis.

Though it seems that several clinical features have frequency differences in the Chinese and French cohorts, only 3 (ptosis, thick eyebrows, and feeding difficulties) show statistical significance that might due to lack of enough Chinese WDSTS samples. Prior to the French cohort study, delayed bone age had been reported in at least 3 patients [6, 17], while significantly advanced bone age was first and only described in a 4-year-old female patient [18]. Here, the French cohort and our cohort reported another 9 and 12 patients presented with advanced bone age and delayed bone age, respectively. Therefore, we propose that both of advanced bone age and delayed bone age are authentic phenotype of WDSTS caused by *KMT2A* variation. Patients 3, 4 and 10 had GH deficiency and also presented with postnatal growth retardation, which are consistent with the results from the French cohort. Moreover, maternity history of Patient 7 supports the surmise of normal fertility in female WDSTS individuals, which has also been reported in two French patients.

The French cohort reported a healthy unaffected father with very low proportion of mosaicism in the *KMT2A* gene (p.Arg1154Trp), who had two affected daughters. Intriguingly, Patient 10 in our cohort was the first WDSTS case reported with mosaicism in the *KMT2A* gene (c.5871 T > A; p.Tyr1957*), who on the other hand presented with more severe phenotypes as well as several previously unreported symptoms (external ear deformity, carpal epiphyseal growth retardation, and dyslipidemia).

Because WDSTS can phenotypic overlap with Pierpont syndrome, Cornelia De Lange syndrome and Kabuki syndrome [6, 7, 9, 19, 20], the candidate genes causing the later three syndromes (i.e. *TBL1XR*1, *NIPBL*, *SMC1A*, *SMC3*, *RAD21*, *HDAC8*, *KMT2*D, and *KMD6A*) in our cohort have been excluded. To date, a total of 72 *KMT2A* variants spanning the whole protein have been identified (including the variants identified in this study), of which 71 are point variants and only one is intragenic large deletion [5–11]. As shown in Additional file 2: Table S2, of the 71 point variants, 48 are truncating, 17 are missense, and 6 are splicing variants. The p.Ser774Valfs*12 variant in Patients 3 and 7 has been previously reported in 3 unrelated patients [11, 21], which qualifies Ser774 as a hotspot for gene variation in *KMT2A*. Other 5 recurrent variants, including p.Arg1154Trp [11], p.Cys1155Tyr [11, 22], p.Gly1168Asp [14, this study], p.Arg1633* [2, 11] and p.Arg2480* [7, 11] were also identified. Although we did not find hotspot variation region, it is noteworthy that only missense variants (7/17) occurred in the cysteine-rich CXXC zinc finger domain (Additional file 2: Table S2), which is predicted to selectively bind to unmethylated CpG-containing stretches of the target genes [10]. Previous studies suggested that patients who harbor the missense variant in the CXXC DNA binding domain may show more severe neurodevelopmental delay [10, 14]. Indeed, in our cohort, Patient 5 (p.Gly1168Asp) has the most severe anomalies in neurological development. In contrast, Patient 4, who has a missense variant (p.Arg3906Cys) in the SET domain showed less neurological abnormalities, although the SET domain plays an important role in transcriptional activation via its H3K4 methyltransferase activity [5]. In the French cohort, 5 patients (Patients 24–28) harbored the missense variants in the CXXC DNA binding domain. 3 of them showed severe ID, while only one patient with severe ID was observed in the other 28 patients. 4 of them had persistent hypotonia (8 in the other 25 patients) and 3 of them had seizures (only one in the other 26 patients). These results further and firmly demonstrate the relationship between dysfunction in the CXXC DNA binding domain and severe nuerophenotypes.

Most of the *KMT2A* variants generate truncated products (the same in Chinese patients, 12/16) that further suggest haploinsufficiency is the main pathogenesis of *KMT2A* gene. It is speculated that missense variants of the *KMT2A* gene are more likely to cause WDSTS by a dominant negative effect [23]. The hypothesis is supported by the fact that upregulation of the *KMT2A* gene transcription in patient with p.Arg1154Trp and site-specific DNA methylation changes driven by *KMT2A* missense variants [9, 10], which might explain the missense variants in the CXXC DNA binding domain linked to a more severe neurophenotypes. Nevertheless, more functional study need be performed to figure out the molecular mechanism, especially why there were only missense variants in this domain.

Currently, it is still difficult to have more conclusions of genotype and phenotype in WDSTS patients due to its broad spectrum of phenotypes. Further studies should attempt elucidating the connection between genotype and phenotype, with epigenotype in consideration. In addition, the clinical interventions for WDSTS patients are yet limited. For most patients, symptomatic treatment, such as rehabilitation training, is the only option to improve the motor development. Results from Patient 3 in our study and another reported 2 patients [6, 16] indicate that GH therapy may be an effective method to improve the height of patients with GH deficiency. Of course, clinical safety should be taken into consideration when using growth hormone therapy.

## Conclusions

In summary, we report 14 Chinese WDSTS patients with 13 pathogenic variants in the *KMT2A* gene, including 11 novel variants. Our detailed comparison between the Chinese and French patients indicates no significant difference in phenotypic spectrum; however, the frequency of several symptoms were different. We demonstrated that variation in the *KMT2A* gene can lead to both advanced and delayed bone age. The novel phenotypes, including macrocephaly, deep palmar crease, external ear deformity, carpal epiphyseal growth retardation, dyslipidemia, and glossoptosis further extended the WDSTS phenotype spectrum. Our study supports the notion that the CXXC zinc finger domain is a hotspot region for missense variants, which is associated with more severe neurophenotypes. In addition, growth curves and endocrine-related problem need further investigation in the future.

## Additional files

Additional file 1: **Table S1.** The variants of *KMT2A* gene in Chinese WDSTS patients. (DOCX 19 kb)

Additional file 2: **Table S2.** Summarize of the *KMT2A* variants in WDSTS patients (DOCX 19 kb)

Additional file 3: **Figure S1.** DNA sequencing results of patient-10. (A) NGS data, (B) Sanger sequencing result. ‘T’ is the wild-type allele and ‘A’ is the variant allele. (TIFF 3994 kb)

## Abbreviations

DD: Developmental delay
GH: Growth hormone
GnRH: Gonadotrophin releasing hormone
H3K4: Histone H3 lysine 4
ID: Intellectual disability
NGS: Next generation sequencing
WDSTS: Wiedemann-Steiner syndrome
WES: Whole-exome sequencing
