# Supplementary material for: Description of the molecular and phenotypic spectrum of Wiedemann-Steiner syndrome in Chinese patients
Source: Orphanet J Rare Dis. 2018 Oct 11;13:178. doi: 10.1186/s13023-018-0909-0 (PMC6180513; doi:10.1186/s13023-018-0909-0)
Supplement: Supplementary file 1 — Table S1. The variants of KMT2A gene in Chinese WDSTS patients. (DOCX 19 kb) [file 13023_2018_909_MOESM1_ESM.docx]

**Table S1.** The variants of *KMT2A* gene in Chinese WDSTS patients.

| Patient | Genomic position  (Chr11_GRCh37:NG_027813.1) | Transcriptional  position (NM_001197104.1)) | Protein  alteration (NP_001184033.1) | Exon/intron position | De novo/  Novel | ACMG Classification |
| --- | --- | --- | --- | --- | --- | --- |
| **This study** | | | | | | |
| 1 | g.118352632 delT | c.3837 delT | p.Pro1281Leufs*75 | Exon 7 | Yes/Yes | Pathogenic |
| 2 | g.118377359 dupA | c.10752 dupA | p.Gly3585Argfs*8 | Exon 27 | Yes/Yes | Pathogenic |
| 3 and 7 | g.118344192 dupC | c.2318 dupC | p.Ser774Valfs*12 | Exon 3 | Yes/No **(ref 11)** | Pathogenic |
| 4 | g.118392684 C>T | c.11716 C>T | p.Arg3906Cys | Exon 36 | Yes/Yes | Likely pathogenic |
| 5 | g.118348850 G>A | c.3503G> A | p.Gly1168Asp | Exon 5 | Yes/**No (ref 14)** | Likely pathogenic |
| 6 | g.118347604 C>T | c.3241C> T | p.Arg1081* | Exon 4 | Yes/Yes | Pathogenic |
| 8 | g.118379917 T>C | c.10900+2 T>C |  | Intron 29 | Yes /Yes | Pathogenic |
| 9 | g.118379852 C>T | c.10837 C>T | p.Gln3613* | Exon 29 | Unknown /Yes | Pathogenic |
| 10 | g.118369153 T>A | c.5871T>A | p.Tyr1957* | Exon 22 | Yes/Yes | Pathogenic |
| 11 | g.118353183 delC | c.4059 delC | p.Pro1354Leufs*2 | Exon 8 | Yes/Yes | Pathogenic |
| 12 | g.118370108 delG | c.6052 delG | p.Glu2018fs*7 | Exon 23 | Yes/Yes | Pathogenic |
| 13 | g.18342775 C>T | c.901C>T | p.Arg301* | Exon 3 | Yes/Yes | Pathogenic |
| 14 | g:118344383 dupC | c.2510 dupC | p.Trp838lfs*9 | Exon 3 | Yes/Yes | Pathogenic |
