# Supplementary material for: Description of the molecular and phenotypic spectrum of Wiedemann-Steiner syndrome in Chinese patients
Source: Orphanet J Rare Dis. 2018 Oct 11;13:178. doi: 10.1186/s13023-018-0909-0 (PMC6180513; doi:10.1186/s13023-018-0909-0)
Supplement: Supplementary file 2 — Table S2. Summarize of the KMT2A variants in WDSTS patients (DOCX 19 kb) [file 13023_2018_909_MOESM2_ESM.docx]

Table S2. Summarize of the *KMT2A* variants in WDSTS patients

| **Amino acid position** | **Protein domain** | **Variants** | | |
| --- | --- | --- | --- | --- |
|  |  | **Truncating** | **Missense** | **Splicing** |
| 1-168 |  | p.Pro51Argfs*84; p.Ser90*  p.Arg160* |  |  |
| 169-309 | AT hook domain | p.Arg301*; p.Glu219Leufs*27 | p.Pro280Thr |  |
| 310-367 |  | p.Val347Leufs*53 |  |  |
| 368-396 | Coiled coil domain | p.Ala383Glyfs*6 |  |  |
| 397-1146 |  | p.Leu717Cysfs*39; p.Arg745*  p.Ser774Valfs*12; p.Gln819*  p.Trp838lfs*9; p.Gln1045Profs*4  p.Arg1081*; p.Arg1083*  p.Arg1101* | p.Ser873Asn; p.Gly1007Cys | c.3334+1G>A |
| 1147-1195 | CXXC zinc finger domain |  | p.Arg1154Trp; p.Cys1155Tyr p.Cys1161Gly; p.Gly1168Asp; p.Gly1181Asp; p.Cys1189Tyr  p.Cys1194Tyr |  |
| 1196-1430 |  | p.Pro1281Leufs*75; p.Pro1354Leufs*2  p.Ser1299Profs*26; p.Gly1338Valfs*18  p.Val1347Trpfs*9 |  | c.4086+1G>A |
| 1431-1627 | PHD domain | p.Lys1534*; p.Cys1556Serfs*2 | p.Cys1448Arg | c.4696+1G>A |
| 1628-1702 |  | p.Arg1633* |  |  |
| 1703-1748 | BROMO domain |  |  |  |
| 1749-1930 |  | p.Pro1868Glnfs*3 |  |  |
| 1931-1978 | PHD domain | p.Tyr1957*; p.Gln1978* | p.His1958Arg | c.5803-1G>A |
| 1979-2017 |  | p.Phe2001Trpfs*8 |  |  |
| 2018-2074 | FYRN domain | p.Glu2018fs*7; | p.Gly2027Glu |  |
| 2075-3665 |  | p.Arg2127*; p.Gln2261*  p.Ser2305Leufs*2; p.Arg2382*  p.Gly2422*; p.Arg2480*  p. Leu2756*; p.Gln2803*  p.Gln2864*; p.Val2936*  p.Glu3448fs*7; p.Gly3585Argfs*8  p.Gln3613*; p.Asp2725Glyfs*31  p.Ile2758Aspfs*2; p.Pro3239Leufs*10  p.Arg2163*; p.Glu2544*  p.Arg2659* | p.Met2853Arg; p.Ser3147Phe  p.Leu3617Pro | c.10900+2 T>C |
| 3666-3747 | FYRC domain |  |  |  |
| 3748-3828 |  |  |  | c.11322-1G>A |
| 3829-3969 | SET domain |  | p.Arg3906Cys |  |
| 3970-3972 |  |  |  |  |

A total of 71 *KMT2A* point variants were identified in Wiedemann-Steiner syndrome patients. 15 Variants are from Chinese patients, including 13 from this study (red) and 2 from a previously reported literature (purple). Variants (black) are previously reported in other ethnic patients.
