# Supplementary figures and images for: Description of the molecular and phenotypic spectrum of Wiedemann-Steiner syndrome in Chinese patients
Source: Orphanet J Rare Dis. 2018 Oct 11;13:178. doi: 10.1186/s13023-018-0909-0 (PMC6180513; doi:10.1186/s13023-018-0909-0)

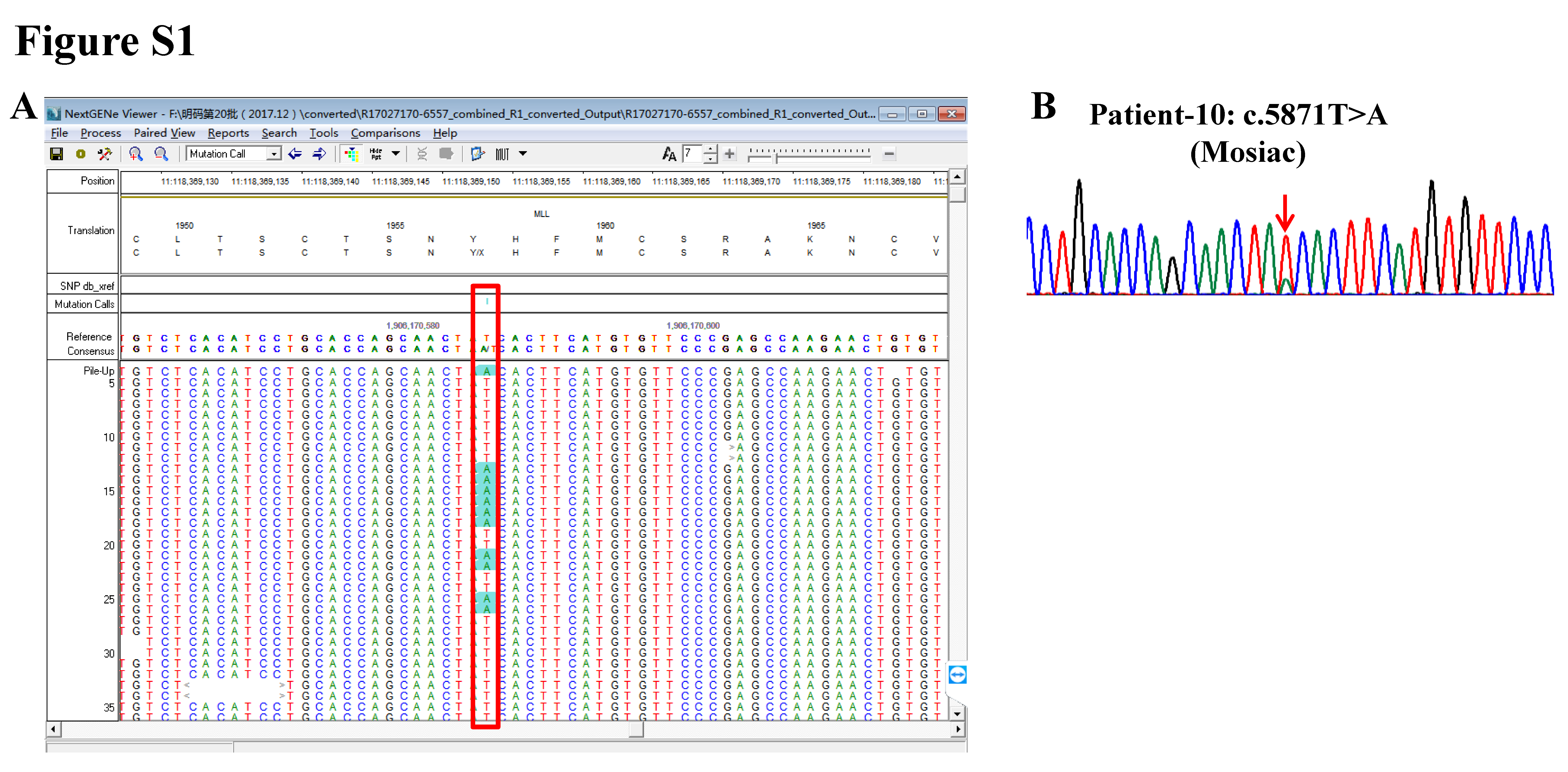

Supplement: Supplementary file 3 — Figure S1. DNA sequencing results of patient-10. (A) NGS data, (B) Sanger sequencing result. ‘T’ is the wild-type allele and ‘A’ is the variant allele. (TIFF 3994 kb) [file 13023_2018_909_MOESM3_ESM.tiff]
